# Effects of different reduction patterns on stress distribution in patients with intertrochanteric fractures with intramedullary nail fixation: a finite element analysis

**DOI:** 10.3389/fbioe.2025.1507774

**Published:** 2025-03-12

**Authors:** Jiajing Zhu, Zhipeng Du, Changpeng Cao, Yang Gao, Xinxiao Chen, Haiyang Xing, Gang Wang

**Affiliations:** ^1^ Department of Radiology, China–Japan Union Hospital of Jilin University, Changchun, Jilin, China; ^2^ Department of Orthopedics, China–Japan Union Hospital of Jilin University, Changchun, Jilin, China; ^3^ The Orthopaedic Medical Center, Second Hospital of Jilin University, Changchun, Jilin, China

**Keywords:** finite element analysis, femur, intertrochanteric femoral fracture, positive cortical support, intramedullary nail

## Abstract

**Objective:**

Positive medial cortical support is a reliable fracture reduction pattern, but existing research on its use is mainly qualitative. We conducted this finite element analysis study to quantitatively evaluate the usefulness of positive cortical support for intertrochanteric fracture reduction.

**Methods:**

Twenty-five models of intramedullary nail fixation for AO type 31-A1.2 intertrochanteric fractures subjected to different reduction patterns were established. The peak von Mises stress at the femoral fracture surface, proximal intersection of the intramedullary nail, and distal intersection of the intramedullary nail, as well as the maximum fracture displacement, were determined by finite element analysis under the three working conditions of standing, walking, and walking stairs.

**Results:**

As the head–neck fragment moved forward, the von Mises stress at the fracture surface, the proximal intersection point of the intramedullary nail, and the distal intersection point gradually decreased. This resulted in reduced fracture displacement, a significant decrease in trabecular bone volume, and a slight increase in the risk of screw cut-out. As the head–neck fragment moved medially, the fracture gained positive support from the medial cortex, leading to a gradual decrease in von Mises stress at the fracture surface and the proximal intersection point of the intramedullary nail, as well as reduced fracture displacement. However, the von Mises stress at the distal intersection point gradually increased.

**Conclusion:**

The reduction pattern involving positive medial, anteromedial, and anterior cortical support may be an effective alternative to anatomical reduction for the treatment of difficult-to-reduce intertrochanteric fractures.

## 1 Introduction

Hip fractures occur with high incidence and mortality rates. It is estimated that by 2025, the number of hip fractures will increase to 2.6 million globally, and by 2050, this number will rise to 6.25 million (Mattisson et al., 2018; Cooper et al., 1992; Gullberg et al., 1997). In 2019 alone, the global number of disability-adjusted life years due to hip fractures was estimated at 2.9 million (Dong et al., 2023). Therefore, hip fractures are considered to have a high economic and medical burden. In the United States alone, the economic loss caused by hip fractures amounts to as much as $7 billion each year. With population aging, the annual cost of care for patients with hip fractures is projected to exceed $16 billion by 2040 (Haentjens et al., 2005).

Intertrochanteric fractures are the most common type of hip fracture, accounting for 55% of proximal femoral fractures (Mattisson et al., 2018; Zhang et al., 2021; Koval et al., 1996; Wu et al., 2021; LeBlanc et al., 2014). Older individuals are the most vulnerable population, and the incidence of intertrochanteric fracture is expected to increase with population aging. In older patients, intertrochanteric fractures are usually comminuted, and anatomical reduction, which is usually the gold-standard treatment option, is often difficult owing to low bone mineral density and structural vulnerability (Jegathesan and Kwek, 2022; Ricci, 2023). In such cases, different degrees of open reduction and the assistance of additional reduction tools are often necessary (Huang et al., 2024). This leads to more complicated surgical steps and longer surgical times (Hao et al., 2023). Reduction patterns other than anatomical reduction are needed to overcome these issues. It is necessary to understand which displacement patterns would achieve good stability when anatomical reduction is not possible. Moreover, as the general condition and medical comorbidities of older patients may restrict the time available for anesthesia, alternative reduction patterns should be achievable within a reasonable timeframe with satisfactory stability.

Previous studies Ling et al. (2022), Chang et al. (2015), Zhang et al. (2019) have proposed fracture reduction with positive medial cortical support. This concept has received substantial attention from surgeons, and numerous studies have shown that the benefits of positive support outweigh the limitations, particularly in terms of biomechanical stability, postoperative recovery, and pain relief (Ling et al., 2022; Chang et al., 2015; Liang et al., 2019; Cho et al., 2018a; Wei et al., 2019). The stability achieved with this intertrochanteric fracture reduction approach provides a suitable mechanical environment for fracture healing, as well as allowing early postoperative exercise, in turn improving functional prognosis (Shao et al., 2021; Cho et al., 2018b). However, existing evidence is mainly qualitative. Quantitative evidence on the usefulness of intertrochanteric fracture reduction with or without positive medial cortical support is scarce.

We conducted this quantitative study to evaluate the effectiveness of positive medial cortical support with the aim of identifying a reduction pattern for intertrochanteric fractures that could serve as an alternative to anatomical reduction in the treatment of difficult-to-reduce intertrochanteric fractures.

## 2 Materials and methods

### 2.1 Patients

Using the hospital’s case system, we excluded patients with femoral deformities, fractures, and bone tumors to select a patient who underwent computed tomography (CT) of the left lower limb for mild soft tissue contusion. The patient was a 26-year-old male, with a height of 170 cm and a weight of 70 kg. CT images of the left femur were acquired at 120 kV and 500 mA with a slice thickness of 0.625 mm. The image data were stored in DICOM (Digital Imaging and Communications in Medicine) format. The study was approved by the local institutional ethical review board (Approval No. 2024102302), and the patient provided written informed consent.

### 2.2 Experimental procedure

The CT images of the femur were imported into Mimics Research 21.0 (Materialise Inc., Leuven, Belgium). Three-dimensional (3D) models of the femur were generated using the threshold adjustment, region grow, and calculate part commands. The 3D models were imported into Geomagic Studio 2013 (Geomagic, US) in STL format for further processing. After defeaturing, hole filling, smoothing, and exact surfacing, a simulation of the femoral cortex was obtained. Due to the variable thickness of the femoral cortex in different regions, we measured the cortical thickness at different parts on the CT images, which ranged from 2 mm (femoral head) to 6.5 mm (femoral shaft). Based on these results, a simulation of the trabecular bone of the femur was obtained using the polygon mesh adjustment command. The cortical and cancellous bone models were imported into SOLIDWORKS 2017 (Dassault Systèmes, US). The overlapping and interfering fields of cortical and cancellous bone were removed to obtain a complete femur model. Thereafter, a standard intertrochanteric fracture model of AO type 31-A1.2 was established by cutting with the segmentation function, with the fracture line extending from the greater trochanter to the lesser trochanter of the femur.

In the femoral fracture model, according to the distance of head–neck fragment displacement in the coronal plane, medial displacement of 5 mm (1-times the cortical thickness) was defined as group ***1***, medial displacement of 2.5 mm as group ***2***, no displacement as group ***3***, lateral displacement of 2.5 mm as group ***4***, and lateral displacement of 5 mm as group ***5***. According to the distance of head–neck fragment displacement along the fracture line in the sagittal plane, posterior displacement of 5 mm (1-times the cortical thickness) was defined as group ***a***, posterior displacement of 2.5 mm as group ***b***, no displacement as group ***c***, anterior displacement of 2.5 mm as group ***d***, and anterior displacement of 5 mm as group ***e*** (Table 1).

**TABLE 1:** Model grouping.

			Coronal plane				
Sagittal plane displacement			Positive		Neutral	Negative	
			5.0 mm	2.5 mm	0 mm	−2.5 mm	−5.0 mm
Sagittal plane	Negative	−5.0 mm	***1a***	***2a***	***3a***	***4a***	***5a***
		−2.5 mm	***1b***	***2b***	***3b***	***4b***	***5b***
	Neutral	0 mm	***1c***	***2c***	***3c***	***4c***	***5c***
	Positive	2.5 mm	***1d***	***2d***	***3d***	***4d***	***5d***
		5.0 mm	***1e***	***2e***	***3e***	***4e***	***5e***

Notes: 1. Medial, lateral, anterior, and posterior displacement refer to displacement of the femoral head–neck fragment relative to the distal femoral fragment. 2. In the coronal plane, positive indicates medial displacement of the femoral head–neck fragment relative to the distal femoral shaft, while negative indicates the opposite. In the sagittal plane, positive indicates anterior displacement of the femoral head–neck fragment relative to the distal femoral shaft, while negative indicates the opposite. 3. Model ***3c*** represents anatomical reduction.

**FIGURE 1:**
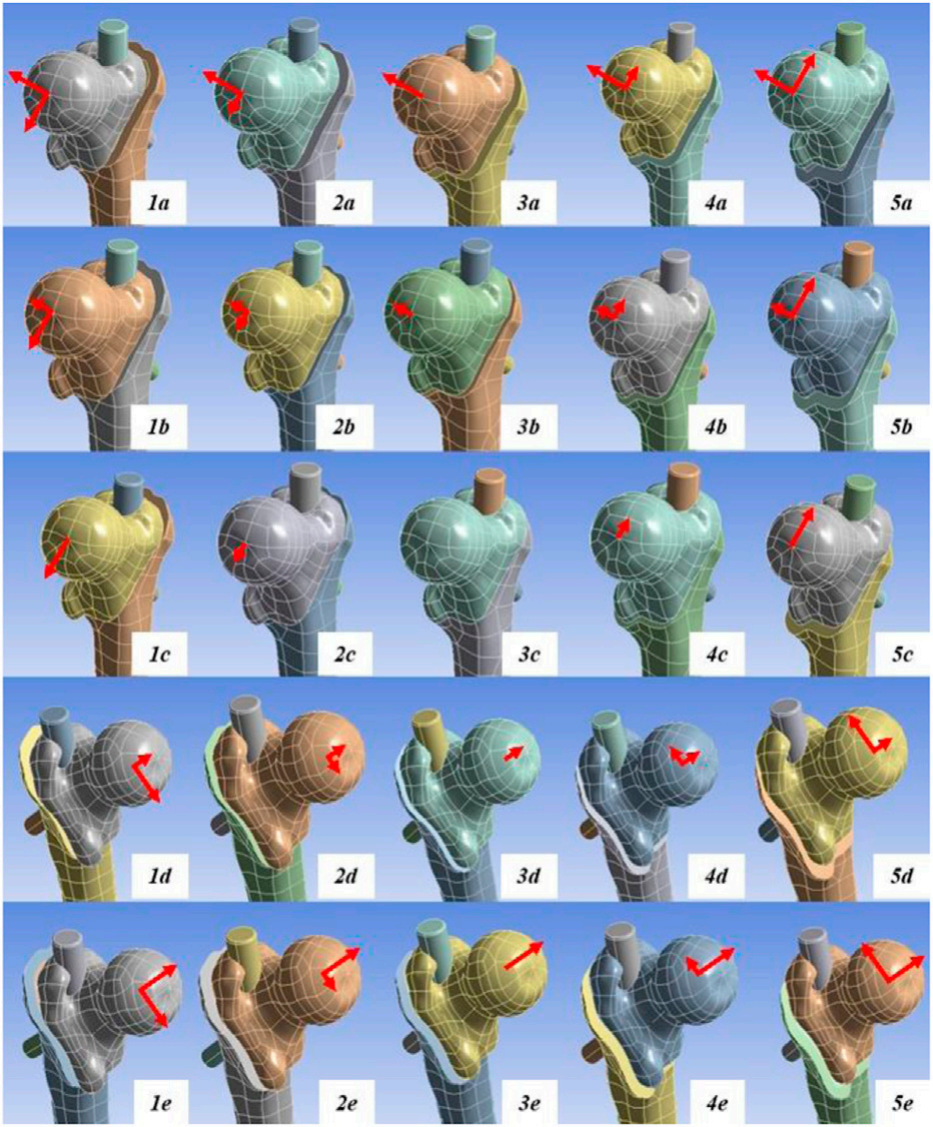
Schematic of the 25 finite element analysis models. Model ***3c*** represents the anatomical reduction pattern. The red arrows indicate the direction and distance of head–neck fracture fragment displacement. The long arrows represent displacement of 5 mm, while the short arrows represent displacement of 2.5 mm.

The 25 fracture models were obtained by matching the five groups of anterior and posterior displacement with the five groups of medial and lateral displacement (5 × 5). The models were saved and exported in Part format for the next step of assembly with the internal fixator. According to the anatomical characteristics of the proximal femoral model and the AO fixation principle, an appropriate Stryker proximal femoral intramedullary nail was selected. The intramedullary nail model was established using SOLIDWORKS 2017 and assembled with the 25 models of intertrochanteric fracture for further finite element analysis (Figure 1).

The finite element models of femoral fracture internal fixation obtained in the previous step were imported into ANSYS Workbench 17.0 (ANSYS, US) for static structural analysis. As we wanted to determine the usefulness of positive cortical support in older individuals with osteoporosis, the model was designed with the same cortical bone and cancellous bone material characteristics that would be observed in the elderly. All materials, including bone and metal, were simplified to isotropic homogeneous linear materials (Mu et al., 2021a; Ma et al., 2022; Li et al., 2021; Polikeit et al., 2003; Wang et al., 2020; Chen et al., 2016) (Table 2).

**TABLE 2:** Material properties.

Model Material	Elastic modulus (MPa)	Poisson’s ratio
Intramedullary nail (titanium alloy)	105,000	0.31
Femoral cortical bone	11,256	0.30
Femoral cancellous bone	197.2	0.28

The contact relationships between the various materials were set according to previous literature (Eberle et al., 2010; Mu et al., 2021b; Sitthiseripratip et al., 2003; Karunratanakul et al., 2013; Goffin et al., 2013). The coefficient of friction between the fracture pieces was set to 0.46, between the intramedullary nail and the femur was set to 0.42, and between the components of the intramedullary nail was set to 0.2. Tetrahedral elements were used to mesh each finite element model. Grid convergence was performed using a 4-mm mesh size, as long as the percent change in the data from the last mesh adjustment was <5%. Finally, the mesh size of the femoral head was determined to be 1 mm, and the mesh size of the other parts of the femur was 3 mm. The mesh size of the intramedullary nail was 2 mm, and the mesh size of the fracture surface and the intramedullary nail surface was 1 mm. After mesh division, the node and mesh numbers of the intramedullary nail model were 87,832 and 53,834, respectively, and of the femoral model were 274,179 and 176,429, respectively (Table 3).

**TABLE 3:** Meshing of 25 three-dimensional models.

Mesh Region	Mesh size (mm)	Node number	Element number
Femur	3	312,820 ∼ 314,769	193,936 ∼ 195,311
Femoral head	1		
Fracture surface	1		
Intramedullary nail	2		
Intramedullary nail surface	1		

Typical working conditions for patients after intertrochanteric fracture surgery include standing, walking, and stair walking (ascending and descending). According to previous literature, the load stress of the hip during standing, normal walking, and stair walking is 100%–140% body weight (BW), 211%–285% BW, and 227%–316% BW, respectively (Bergmann et al., 2001). To simplify the calculation, the following three working conditions were established: 1) in the standing group, the femoral load was 100% BW vertically downward, with 15° hip adduction; 2) in the walking group, the femoral load was 250% BW vertically downward, with 10° hip adduction, 20° flexion, and 15° external rotation; and 3) in the stair walking group (descending), the femoral load was 300% BW vertically downward, with 10° hip adduction, 10° flexion, and 15° external rotation (Mu et al., 2021b) (Table 4).

**TABLE 4:** Mechanical loading in the finite element analysis.

Position	Load	Adduction	Flexion	External rotation	Torque force
Standing	700 N	15°	-	-	-
Walking	1750 N	10°	20°	15°	7 N⋅m
Stair walking (descending)	2100 N	10°	10°	15°	7 N⋅m

**FIGURE 2:**
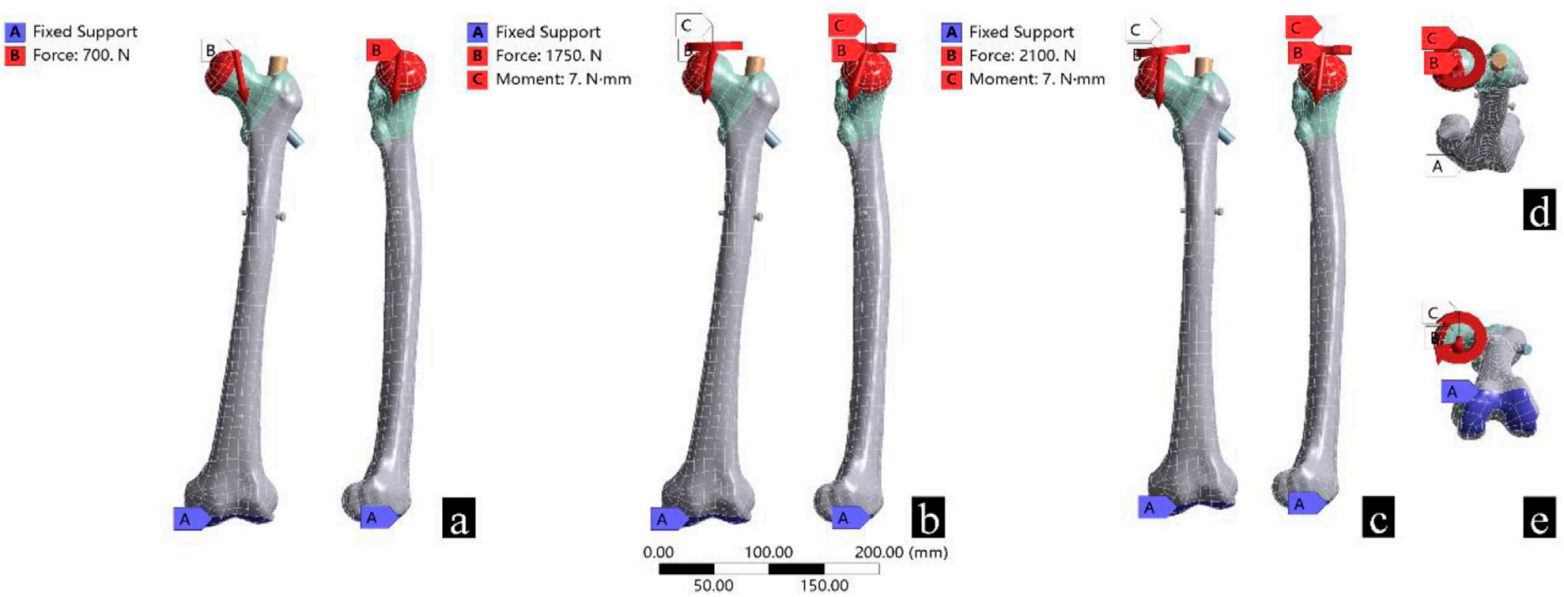
Boundary conditions and loads for the three different working conditions. **(a)** Represents standing, **(b)** represents walking, **(c)** represents stair walking, (**d**) shows the torque loading, and (**e**) shows the location of the fixed support.

According to previous literature, the femur bears different degrees of torsional force during weightbearing. Therefore, 7 N m torque force was applied to the walking group and the stair walking group (Mu et al., 2021b; Schneider et al., 2001; Duda et al., 1997). Coordinate systems for standing, walking, and stair walking were created using the ANSYS coordinate system. The contact surface between the femoral head and the acetabulum was considered as the loadbearing surface, and the distal articular surface of the femur was used for fixation (Figure 2).

### 2.3 Observation indices

Based on our clinical experience and the existing literature (Wu and Tang, 2019; Abram et al., 2013; Jiang et al., 2023; Zhan et al., 2021; Zhan et al., 2024), the following indicators were selected to comprehensively evaluate the biomechanical performance of different reduction models while avoiding numerical singularities: the von Mises stress peak on the femoral fracture surface and at the proximal and distal intersections of the intramedullary nail, the maximum fracture displacement, the yield rate, the cut-out risk, and the average stress of femur models.

## 3 Results

**FIGURE 3:**
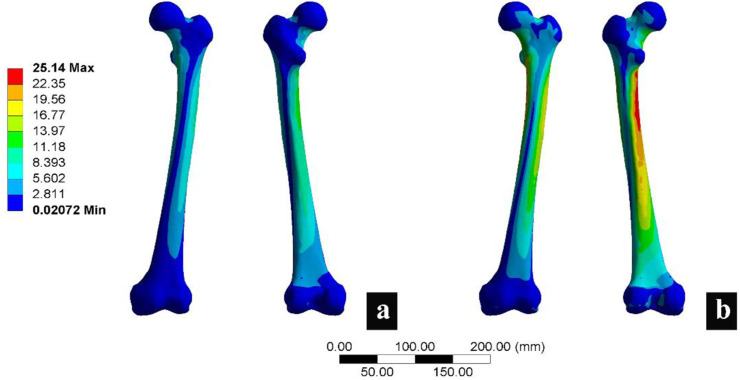
Femoral finite element analysis verification results. **(a)** Represents the calculation results under a load of 750 N, and **(b)** represents the calculation results under a load of 1500 N.

To validate the reliability of the finite element analysis, 3D model morphology measurements and finite element analysis were conducted. The morphological measurements of the 3D model closely matched the measurements of the original CT images (Supplementary Table S1). The finite element analysis results included measurements of the maximum stress of the femur (Cun et al., 2020; Zhang et al., 2024) (Figure 3; Supplementary Table S2) and proximal femoral strain (Li et al., 2024) (Supplementary Table S3), which were consistent with previously published studies. These findings confirmed the reliability of the model.

**FIGURE 4:**
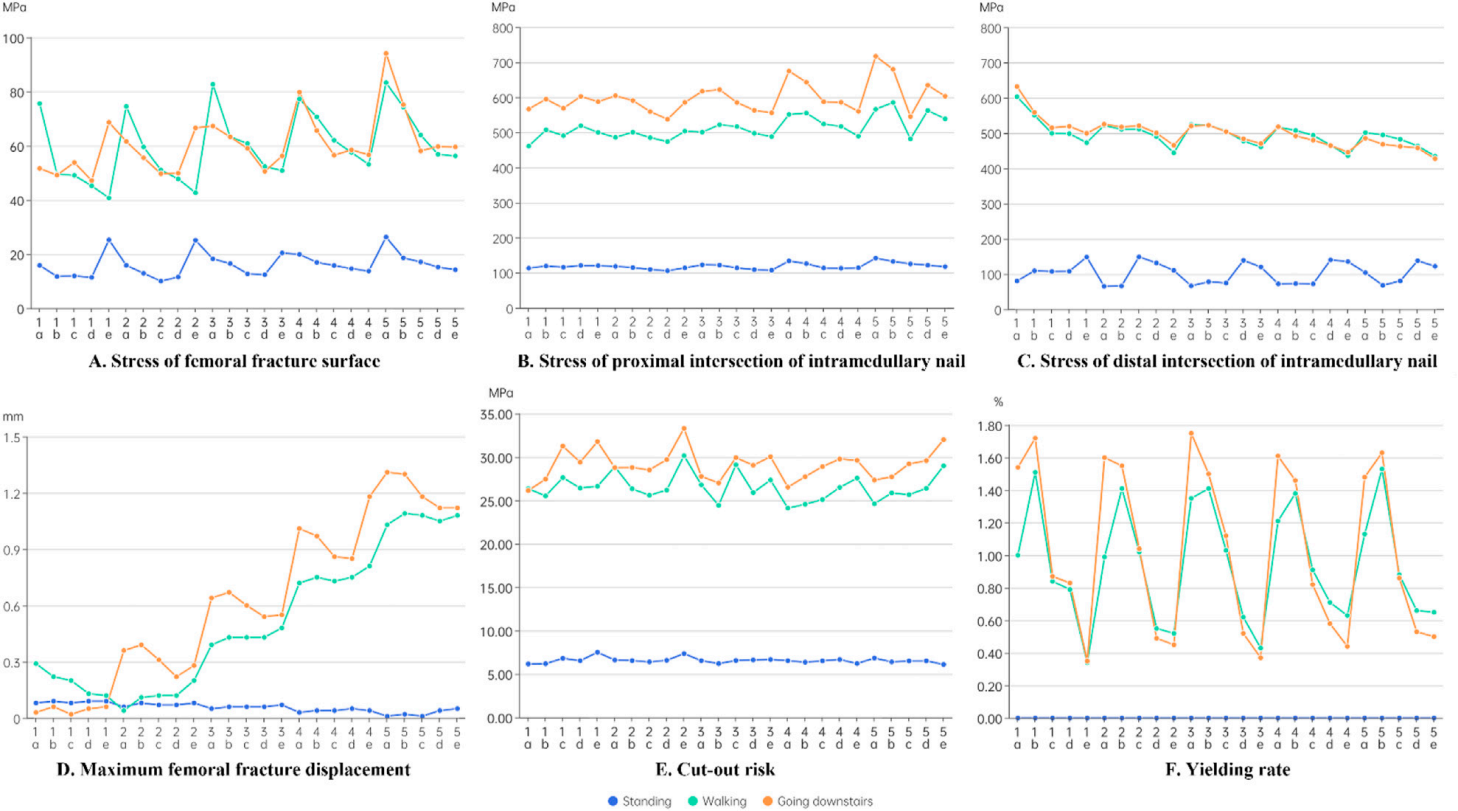
Results of the finite element analysis. **(A)** Stress of femoral fracture surface. **(B)** Stress of proximal intersection of intramedullary nail. **(C)** Stress of distal intersection of intramedullary nail. **(D)** Maximum femoral fracture displacement. **(E)** Cut-out risk. **(F)** Yielding rate.

According to Figure 4, the peak von Mises stress was significantly lower at the fracture surface than at the intramedullary nail. The von Mises stress at both the fracture surface and the intramedullary nail was notably higher during walking and stair walking (descending) than during standing. Figure 4A shows that the von Mises stress at the fracture surface decreased gradually from “***a***” to “***e***”, while it increased from “***1***” to “***5***”. This trend suggested that the anterior and internal positioning of the head–neck fragment reduced the stress that could be endured by the fracture surface, thus promoting a favorable mechanical environment for fracture.

Figure 4B shows that the stress at the proximal interlocking region of the intramedullary nail increased as the complexity of the loading condition increased, with walking and stair walking (descending) producing significantly higher von Mises stress than standing. Another trend was that the stress at the proximal interlocking region decreased progressively from “***a***” to “***e***”, while it increased from “***1***” to “***5***”.

Figure 4C shows that the stress at the distal region was comparatively lower. Additionally, the stress at the distal interlocking region decreased progressively from “***a***” to “***e***”, and from “***1***” to “***5***”. This indicated that the anterior and external positioning of the head–neck fragment reduced the stress at the distal intersection of the intramedullary nail.

According to Figure 4D, during standing, there was no consistent pattern of fracture displacement across the 25 models. However, during walking and stair walking (descending), fracture displacement significantly increased from “***1***” to “***5***”, and it decreased from “***a***” to “***e***”. This suggested that external and posterior positioning of the head–neck fragment may have reduced the stability of fracture alignment. Moreover, anterior and posterior movement of the head–neck fragment had a more obvious influence on the stability of the fracture line than internal and external movement.

According to Figure 4E, with an increase in load, the stress on the proximal locking screw also increased. The overall trend showed a gradual increase from “***a***” to “***e***”, while no significant pattern was observed from “***1***” to “***5***”. This suggests that anterior displacement of the head–neck fragment may increase the risk of screw cut-out, but the effect was not significant. Furthermore, anterior and posterior displacement of the head–neck fragment had a more noticeable impact on the risk of screw cut-out than internal and external displacement.

According to Figure 4F, in the standing condition, the load was too small to obtain data on trabecular bone yield. In the other two conditions, the yield rate of groups ***a*** and ***b*** was significantly higher than that of groups ***d*** and ***e***, with group ***c*** falling in between. This trend suggests that posterior displacement of the head–neck fragment significantly increased the yield rate of trabecular bone, while internal and external displacement of the head–neck fragment had a less noticeable effect on the yield rate.

**FIGURE 5:**
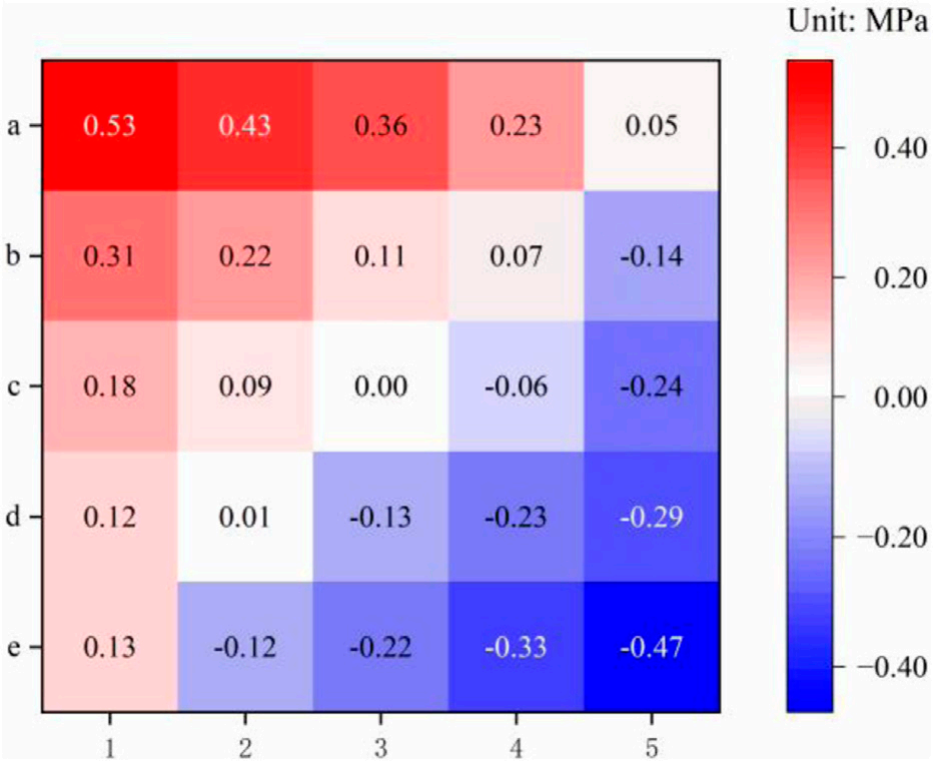
Average stress of each of the 25 models of femoral fracture.

Figure 5 shows the trend in the average stress distribution for the 25 femoral fracture models relative to the anatomically reduced model. A very clear pattern can be observed, showing that compared to anatomical reduction of the head–neck fragment, medial displacement (***5→1***) and posterior displacement (***e→a***) led to an overall increase in femoral stress, while lateral displacement (***1→5***) and anterior displacement *(* ***a→e***) led to a decrease in overall femoral stress.

## 4 Discussion

Anatomical reduction remains the gold-standard open reduction fracture treatment. However, for older patients with intertrochanteric fracture, anatomical reduction is difficult. Furthermore, the osteoporotic bone fixation efficiency in aged people is lower than in younger people, which may result in unsatisfactory reduction and post-surgical displacement. Anatomical reduction by closed manipulation is difficult to achieve in patients with intertrochanteric fracture. Many factors interfere with anatomical reduction, including the irregular anatomical contour in the metaphyseal transition area, eccentric gravity conduction, poor reduction and fixation of the lesser trochanter, and the limited resolution of intraoperative X-ray fluoroscopy (Wei et al., 2019). Therefore, reduction with positive cortical support may be a useful alternative to anatomical reduction in clinical practice.

The results of this study show that as the head–neck fragment moved anteriorly (***a*→*e***), the fracture received positive support from the anterior cortex, and the von Mises stress at the fracture surface, the proximal intersection of the intramedullary nail, and the distal intersection of the intramedullary nail gradually decreased. This led to a reduction in fracture displacement, a significant decrease in trabecular bone yield, and a slight increase in the risk of screw cut-out. Meanwhile, as the head–neck fragment moved medially (***5*→*1***), the fracture received positive support from the medial cortex, and the von Mises stress at the fracture surface and proximal intersection of the intramedullary nail, as well as fracture displacement, gradually decreased, while the von Mises stress at the distal intersection gradually increased. As the loading conditions became more complex, these trends became more pronounced.

From the perspective of fracture healing, moderate mechanical stimulation in the early stages of healing accelerates callus formation, especially with fractures that predominantly heal through indirect mechanisms, such as is the case with intertrochanteric fractures. However, excessive compressive stress can hinder fracture healing (Travascio et al., 2021). The findings of this study indicate that positive anterior, medial, and anteromedial cortical support reduced stress at the fracture site and provided a more stable initial environment for healing (Figures 4A, D). Mao et al. (2023) reached the same conclusion, showing that after the fracture received medial cortical support, the stress on the fracture surface decreased. Another previous study showed that fracture displacement by >2 mm had adverse effects on fracture healing, whereas displacement by <0.5 mm improved fracture ossification (Kenwright and Gardner, 1998). None of the 25 models evaluated in the present study showed maximum displacement of >2 mm (Figure 4D), and no adverse effects on fracture healing were observed under any of the three loading conditions. However, from the perspective of fracture fixation stability, as the head–neck fragment displaced laterally and posteriorly, the fracture progressively lost strong support from the medial and anterior cortices, leading to a significant increase in fracture displacement and resulting in unstable fixation (Figure 4D). This finding is consistent with previous research. Shao et al. (2021), in their finite element analysis of an intertrochanteric fracture model with lateral wall failure, found that negative support from the anterior cortex and medial cortex increased the relative displacement between fracture surfaces. Additionally, negative support from the anterior cortex significantly increased the yield rate of trabecular bone, whereas positive support had the opposite effect (Figure 4F). Yielding is the process in which permanent deformation occurs when the stress on a material exceeds its elastic limit. In fracture fixation systems, yielding is observed when the stress on bone tissue exceeds its maximum tolerance, leading to deformation or collapse. As the yield rate increases, the stability of fracture fixation gradually deteriorates, potentially leading to loosening, fatigue, and ultimately fixation failure (Qu et al., 2009). Therefore, the anterior and medial positioning of the head–neck fragment is significantly related to fracture stability. Cho et al. (2018a) also demonstrated that positive support from the medial cortex increased the stability of fracture fixation. They found that patients with positive support from the medial cortex had a significantly shorter screw migration distance than those with negative support from the medial cortex (P < 0.05). From the perspective of fracture healing and fixation stability, positive anterior and medial cortical support is recommended as a preferred method for reducing difficult-to-reduce intertrochanteric fractures (Cho et al., 2018a).

**FIGURE 6:**
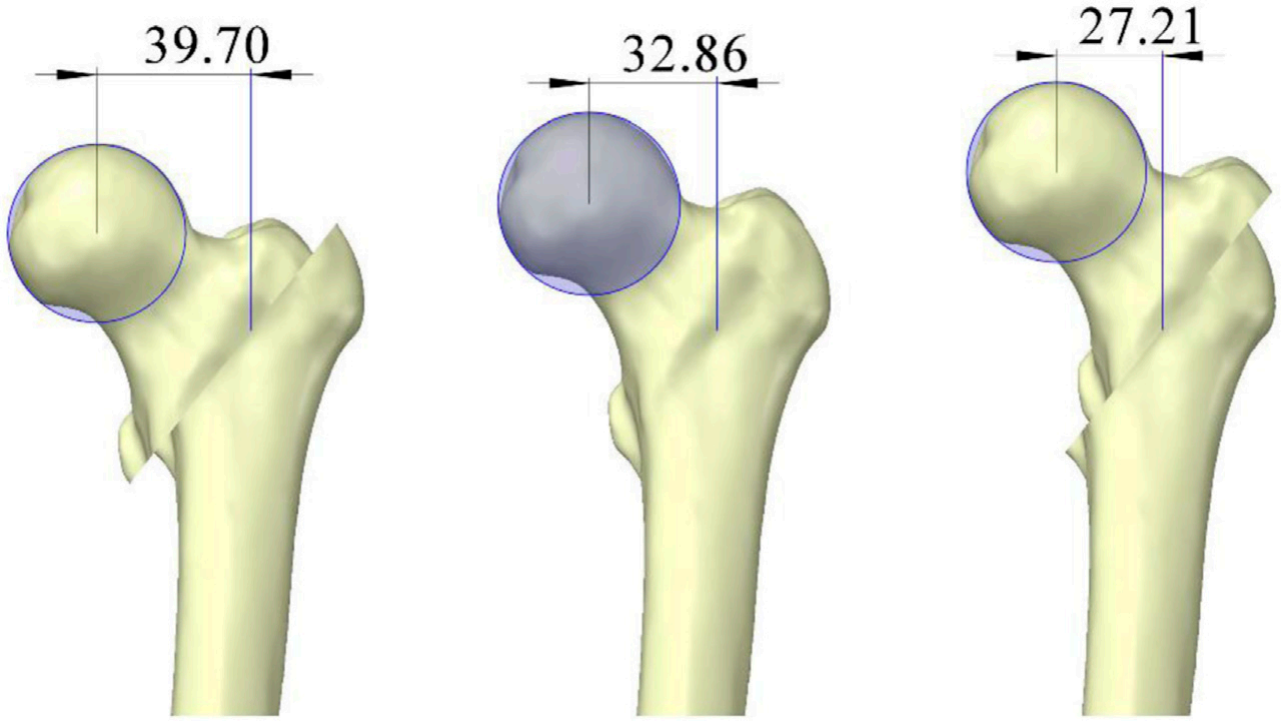
Lever effect caused by changes in the position of the femoral head and neck fracture block (units: mm).

From the perspective of stress at the intramedullary nail, the stress at the proximal cross-section exceeded 450 MPa in all models during both walking and stair walking (descending) (Figures 4B, C). Irreversible deformation can occur when the stress of the titanium alloy internal fixator reaches 450 MPa, and the internal fixator may become fissured and broken when the stress reaches 600 MPa (Vajgel et al., 2013). This suggests that regardless of the reduction method, complex postoperative recovery activities should be avoided in older patients with osteoporosis during the early postoperative period. Additionally, a clear trend was observed in that the head–neck fragment was displaced anteriorly (***a→e***) and the proximal femur transitioned from negative to positive anterior cortical support, resulting in reduced intramedullary nail stress. Shao et al. (2021) reached the same conclusion. They analyzed nine different reduction methods based on the pairwise combinations of positive, neutral, and negative cortical support and found that the positive–positive cortical support reduction method resulted in the lowest intramedullary nail stress, while the negative–negative support reduction method led to the highest intramedullary nail stress. This means that both negative support from the medial cortex and negative support from the anterior cortex led to higher stress being borne by the intramedullary nail. The same experimental results were reported by Mao et al. (2023). After positive medial cortical support was applied, the stress on the intramedullary implant significantly decreased. With medial positioning of the head–neck fragment (***5→1***), the proximal femur transitioned from negative to positive medial cortical support, leading to decreased stress at the proximal cross-section of the intramedullary nail and increased stress at the distal cross-section. We attribute this opposing trend to a lever effect (Figure 6). When positive medial cortical support is achieved, the lever arm formed by the proximal locking screw and the main nail increases in length, increasing the moment. Given the limited space available for the proximal locking screw to move, more force is transmitted along the main nail to the distal cross-section of the intramedullary nail. Conversely, when the medial cortex is under negative support, the entire lever arm decreases in length, reducing the moment, and more force is concentrated at the proximal cross-section of the intramedullary nail. Clinical observations have also shown that the proximal cross-section of the intramedullary nail is the area most prone to wear and fracture (Wu and Tang, 2019), making positive medial cortical support a preferential reduction method.

By combining Figures 4, 5, it can be observed that when the head–neck fragment was positioned anteriorly (***a*→*e***), the femoral proximal region transitioned from negative anterior cortical support to positive anterior cortical support. As a result, the overall femoral stress decreased, and the stress at both the proximal and distal intersections of the intramedullary nail was also reduced. This suggests that positive anterior cortical support alleviated the stress load on the entire fixation system. In other words, under constant loading, a reduction in overall stress suggests a more even stress distribution. Jiantao et al. (Li et al., 2019) suggested that unstable fractures are usually accompanied by an incomplete posterior intertrochanteric wall or lateral wall fracture. Unstable intertrochanteric fractures are usually accompanied by dissociation of the lesser trochanter as a posterior medial wall defect, and reliable posteromedial support for stable reduction is difficult to achieve in those with posterior displacement. Studies have proven that the probability of loss of support after surgery is as high as 80% in patients with negative anterior cortical support (posterior displacement of the head–neck fragment) during intraoperative reduction (Chang et al., 2018). Therefore, positive anterior cortical support is the recommended reduction method for difficult-to-reduce intertrochanteric fractures.

As difficult-to-reduce intertrochanteric fractures in older individuals are highly complex and challenging to simulate, this study only modeled AO type 31-A1.2 intertrochanteric fractures and inferred trends in internal fixation outcomes. However, in clinical practice, difficult-to-reduce intertrochanteric fractures are usually unstable, and they may be accompanied by fractures of the greater trochanter, in which the entry point of the intramedullary nail is mostly incomplete (Li et al., 2019; Evans, 1949). Additionally, such fractures are often accompanied by defects and comminution in the posteromedial wall. Therefore, the predictions of this study are conservative, and the same reduction methods may result in poorer mechanical outcomes when used in clinical practice, although this remains to be investigated.

This study has several limitations. First, a single bone model was used, which may affect the generalizability of the results. Second, to simplify the computational model, the femoral material was simplified to a homogeneous and isotropic material, which may affect the accuracy of the model’s predictions under complex loading conditions. Finally, a plane osteotomy method was used to simulate the fracture, which may have introduced some discrepancies between the results of this study and real-life fractures. Therefore, these results should be validated with biomechanical testing and clinical outcomes.

## 5 Conclusion

This study explored the rational application of positive cortical support for the reduction of intertrochanteric fractures in the elderly. The results indicate that positive support from the anterior medial, anterior, and anteromedial cortices can provide stable fixation and create a favorable biomechanical environment, suggesting that these support patterns may serve as effective alternatives to anatomical reduction. This study not only quantitatively analyzed the feasibility of using positive cortical support in the treatment of intertrochanteric fractures, but it also provided theoretical evidence for treating difficult-to-reduce intertrochanteric fractures. However, further clinical trials are necessary to assess the broader applicability of anterior medial, anterior, and anteromedial cortical support as reduction methods in clinical practice.

## Data Availability

The raw data supporting the conclusions of this article will be made available by the authors, without undue reservation.
